# American Dental Association

**Published:** 1885-01

**Authors:** Chas. Mayr


### AMERICAN DENTAL ASSOCIATION.

TWENTY-FOURTH ANNUAL MEETING, AT SARATOGA.

REPORTED FOR THE INDEPENDENT PRACTITIONER BY PROF. CHAS. MAYR.

(Concluded from page 699, Vol. V.)

The consideration of the report of Section VII, Physiology and Etiology, was declared in order.

*Dr. Pierce*—Has watched the development of the various theories regarding dental caries, and especially the experiments of Dr. Miller. He does not think that Dr. Barrett comprehends the position that Dr. Miller occupies. He proceeded to read extracts from the papers of the latter, in which he speaks of outside influences, and the existence of hidden causes aside from those of a bacterian origin. He thought these causes were antecedent to absolute decay, and that Dr. Miller would imply that the diseased condition existed first, and that bacteria, finding here a field prepared for their growth, multiplied, not as the cause, but as the consequence of impaired function and nutrition. An impairment of the vital forces is the first step; the diseased condition being established, bacteria find then the proper environments for their growth.

*Dr. Barrett*—No one more than myself regrets the absence of Dr. Miller, or my own inability to properly and intelligently present his views. No one who has not himself gone through a series of experiments and original observations sufficient to enable him to thoroughly comprehend the matter, is competent to speak knowingly on this subject. He may give an opinion, but his facts are all derived from some one else; everything except his mere theoretical speculation is second-hand; he is liable to quite misapprehend the whole matter, because of the necessity for looking through the eyes of another. No one who is not an original observer possesses the right to express himself positively. For these reasons I am exceedingly chary of making a definite statement; but I think I am quite warranted in asserting that Dr. Pierce could not be wider of the mark in his apprehension of what Dr. Miller teaches. He seems to be under the impression that the latter regards micro-organisms as only incidental to caries. Dr. Miller asserts directly the contrary, and it would seem that any one who reads his paper as intelligently as Dr. Pierce must necessarily do, could come to no other conclusion than that his observations and experiments indisputably establish that the fungi have an active influence in directly promoting decay. He says, indeed, that there are doubtless other factors, some of which he has determined, and these he proposes to investigate. The limits of research have not yet been reached; there are yet higher hills to climb before we shall be able to see the end. But the theory propounded by certain microscopists that micro-organisms are simply the scavengers of filth, the destroyers of effete matter, is not the theory of Dr. Miller, nor do I think he would, for a moment, assent to such an interpretation of that which he has written and said.

*Dr. Daboll*—Those who labor under the impression that Dr. Miller does not give due credit to all who have made any original investigations are much mistaken. To Magitot, to Underwood and Milles, and to all who have studied the subject for themselves, Dr. Miller gives all attention, even while differing from them. But those who, on the strength of mere theoretical speculation, misinterpret the work of observers, and endeavor to wrest demonstrated facts to the support of a preconceived hypothesis, he does not deem worthy a serious answer.

*Dr. Atkinson*—I am justified in saying that we have a ghost; an unseen current of nutrient activity is the first stage of the movement which assists in bringing about the process of decay.

*Dr. Searle*—I have read Dr. Miller's essays critically, and understand him to say that there exists a chemical union between the lime-salts and the basis substance of the dentine. This union is brought about by vital forces, and in certain cases of ill health these forces break it down.

*Dr. F. Y. Clark*—I think that the bacteria produce caries of teeth by the absorption of the bioplasson, or living substance in the meshwork of the teeth. I suppose there are life-cells and death cells—we may call the bacteria death-cells—and that there is a continuous fight between the two sets of cells. Tyndall says there are classes of organisms which live directly upon the living tissue, and the same organisms, when the tissue is inflamed, feed upon the products of the inflammation ; I do not see how Dr. Miller has produced that condition in any of his experiments.

After considerable desultory discussion, the subject was finally passed.

SECTION I ARTIFICIAL DENTISTRY, CHEMISTRY AND METALLURGY.

Dr. William H. Trueman, chairman, presented the report of the Section. The paper was very comprehensive, but the reporter was unable to follow it sufficiently to present a faithful abstract.

Dr. John Allen read a paper upon Prosthetic Dentistry. In the construction of artificial dentures, he said, we should carefully study the various requirements of different patients, for no two cases are alike; we wish to reproduce organs which nature, with all her skill, has made; the height of art will be to conceal it. This, together with their practical utility, should be the great point to attain, and this requires skill and the perfection of artistic manipulation, together with thorough mental training and scientific research. Hence the impropriety of calling this branch of our profession mechanical dentistry.

According to the best lexicographers, a mechanic is one who pursues a mechanical trade according to definite rules, and the product of whose skill reaches the same result in all cases. But a skillful dentist avoids this mechanical sameness; he has never two cases which require the same procedure; consequently this branch of his professional duty pertains to the artist whose power of perception enables him to see what is necessary to meet the various exigencies involved in his operations.

To accomplish perfect results he must know the nature and properties of the various metals and minerals used in dental practice, together with the art of converting them into artificial dentures. Especially is this the case if he does continuous gum, or block work. Many dentists do not possess these qualifications. We come much nearer the acme of our ambition in continuous gum-work than by any other of the various bases.

*Dr. Robinson*—This section has a very wide range, and is particularly fitted, according to the law of evolution, to survive. I take the words of Pope for my motto :

> "Be not the first by whom the new is tried,
> Nor yet the last to throw the old aside."

For forty-eight years I have labored in the profession. It is proper to inquire what qualifications are requisite for the performance of the several duties of a dentist. Simple mechanical proficiency does not constitute a good dentist; there is poetry and art in the appearance of the work, and science in the adaptation and articulation. These combined, constitute the philosophy of the dentist.

Natural teeth are never oblong or round. All teeth have a peculiar shape ; to give to artificial dentures a natural appearance, they must conform to natural forms. The true artist finds a perennial glory in his labor and imagination, for labor is only the visible form of imagination; anything short of it never fills the whole mind, but only a superficial part. The real artist is never satisfied with what he does occasionally from good impulses. Without the scientific principle, all labor bestowed upon teeth is only elaborate nonsense, for they never satisfy the users.

We find in natural physiognomy teeth which do not agree with the face, but that is only when nature has been obstructed. It is seldom that good and perfect teeth, however plain, can be made better, without destroying the character of the face.

It is the combination of artist and mechanic that makes a good dentist. One of the essential qualifications for a dentist is to be able to make his own instruments, and to do his own work. Even with the variety of instruments in the market, they are so easily broken that we cannot get along without being able to do our own repairing, and hence the mechanical dentist should be a thorough mechanic.

A great charm of men and professions lies in moral rectitude. This higher perception is growing in the profession, as the profession is growing in the community. It will no longer be necessary to extract the teeth of a child, if the parents can be induced to bring the child early; with a disk we can easily polish away the first decay.

Most of the pain in excavating can be prevented if equal parts of caustic potash and carbolic acid be applied to the cavity before cutting. The carbolic acid coagulates the albumen in the tubuli, and both are antiseptics, and harmless to the bone tissue. If the pulp is exposed, the sloughing is instantly removed. The bacterian theory must now be considered as an accepted fact, and the antiseptic treatment presupposes the putrefactive process of disease.

When the chlorine contained in the buccal fluids comes in contact with tin, it is set free and forms chloride of tin, which is antiseptic ; thus tin becomes the best material for preservation. Amalgam fillings are failures when the cavity is below the margin of the gums. They are neither antiseptic nor escharotic. There is something as much beyond microscopic examination and chemical experiment as being is beyond existence, and as spirit is beyond matter. Tin is difficult to pack into a cavity, and the best form for it is that of fine fibers. For delicate margins it is the best possible material; when combined with platinum, it is more ductile and harder; when combined with a little gold, it becomes harder still. The true artist is never selfish; he is serving his calling in restoring and recovering what is lost, and that is the reason why many of our best operators are so poor.

The subject was passed and Section II was called.

### DENTAL EDUCATION.

The Chairman, Dr. C. N. Pierce, read the report, which closed by the presentation of resolutions providing that all students of dental colleges should attend at least two full courses of lectures in different years; that a proper preliminary examination should be demanded; that a proper graduating examination should be required. The report expressed gratification at the recent results obtained by the meeting of the faculties of the dental colleges. Of the three hundred and sixty-five students graduated in the last five months from the twelve principal schools, five per cent, or eighteen, will, by the end of the first year, have dropped out from the profession. At the end of the second year as many more will have sought other occupations; not more than fifteen will be actually interested in the profession at the end of the fifth year.

*Dr. Spaulding*—Is gratified with the results obtained. He only objects to that clause in the agreement of the schools that admits men holding medical degrees to become authorized practitioners in dentistry, by attendance upon but one course. A man who is only a graduate in medicine is not as well fitted, not as competent to become a skillful dentist in one course, as the man who is a good chemist and skillful mechanic. We have catered too much to the medical profession.

*Dr. Hunt*—Expressed gratification that so long a step has been made toward harmonious action by the colleges.

*Dr. Friedrichs*—Disagrees with the view that graduates of medicine ought not to be granted special privileges. When a man graduates an M. D., he has the right by that authority to practice, if he feels like it, and the action of the college faculties is wise. It is well to offer inducements for thorough education.

*Dr. Allport*—Thanked the section for the report, more especially for that particular part referring to practitioners in medicine. It has long been a favorite theory with him that a practitioner in dental surgery should be a specialist in medicine, and that for the treatment of any organ of the body his knowledge should be founded on general medicine. He would like to demand the degree of M. D. for all dentists, not for the sake of the degree itself, but for the education involved in it. He does not like to have dentistry called a profession, for it is properly a specialty of the medical profession. Only as far as it is medical it is a profession ; take that from it, and it becomes merely mechanical.

*Dr. Spaulding*—Let us first educate our students in dentistry, and lay a scientific foundation for practical dentistry, and then, if they choose to adorn themselves with the medical degree, so much the better.

*Dr. Allport*—We have no text-books, even for the foundation of our profession. (Dr. Atkinson: Thank God !) We must go for them to the medical profession.

*Dr. Abbott*—Thinks it plain, from what has been said, that all mean about the same thing. General anatomy is the foundation of both sciences, and the same is the case with general physiology. The questions treated in physiology are not medical, but scientific. No man can get any too much education; so the more we demand, the more we ask of young men, the better for them when they have grown up.

*Dr. Rhein*—If the works on anatomy, physiology, and chemistry are not medical, what then are medical works ?

*Dr. Atkinson*—Every example of a medically educated man who claims to be a better dentist is a subterfuge and falsity. I was educated a medical man, and had to cut loose from it before I considered myself fit to be a healer, and thus with all who are not educated in hospitals. A mere matter of lectures does not make men fit to be healers. Let us not fool away time and glorify the old embryonic position. I am thankful that we have no text-books; surgery is ahead of medicine everywhere. Look at medicine, which has high dilutions and low dilutions, allopaths, homœopaths, and no-paths. Through what would a man have to go? First, classical education, then medical education, then a matriculation in a dental college. But before that I say he must be well versed in the use of his fingers. Some of the greatest fools are the most respected in society, and the most assuming are the least educated. If there is a man who is exclusive, it is the ignoramus who thinks he knows all. Many of these men have false degrees, ornaments of sin. I want no such ornament.

The subject was passed, and the society adjourned.

DENTAL LITERATURE AND NOMENCLATURE.

Upon the opening of the evening session of Thursday, Section III was called. The report (which was incomplete, but will be presented in full in the official proceedings) was read by the Chairman, Dr. J. Taft. He reviewed the third edition of Prof. Garretson's system of Oral Surgery, and raised a warning against the severe strictures of amateur critics.

*Prof. Mayr*—Presented a few facts about foreign journals. He asserted that Austria, with its forty millions of inhabitants, has no dental journal. In Spain a journal is issued with the "approval of the bishop." The French dental journals are not up to the standing of the nation in other respects.

OPERATIVE DENTISTRY.

Section IV was called. The report was read by Dr. E. T. Darby, Chairman, who presented four subjects for discussion. The Herbst method of packing gold by revolving burs; the electric incandescent light, its uses in dental practice; the combination of gold and some baser metal; the danger of excessive dryness during long operations while employing the rubber dam, thereby causing shrinkage of the tooth substance, and subsequent expansion, thus causing the filling to leak.

The discussion was mainly upon the Herbst method of packing gold. Drs. Abbott, Hunt, and Daboll gave their experiences, which were limited, and mostly from reports.

*Dr. Watkins*—Had a tooth filled in his own mouth by that method, the operator using agate burnishers, made at the suggestion of Dr. Wheeler, of Albany.

*Dr. McKellops*—Had procured a set of instruments, but had not been able to produce satisfactory results.

*Dr. Barrett*—Had a limited experience. He described the Wolrab gold, which is very soft. The instruments are not polished, only smoothed, and the gold is used in the form of cylinders. Dr. Herbst keeps a piece of block tin on his operating table, upon which he rubs his instruments, and thus keeps them free from adhering gold.

*Dr. Hunt*—Had inserted fillings by the rotary process, using Williams' cylinders with fair success. If the burnisher be continuously pressed upon the gold, an uncomfortable degree of heat is evolved by the friction. The pressure should be interrupted.

*Dr. Rhein*—Thought that the coherence of the soft cylinders was brought about by the heat produced by friction, which annealed the gold.

*Dr. Daboll*—Said that it was absurd to suppose that an annealing heat could be produced in the cavity without torturing the patient beyond forbearance.

*Dr. Barrett*—Said that in some as yet unexplained manner, exceedingly soft gold that under the impact of the mallet could not be worked into a coherent mass, was, by the rotary process, made so solid and homogeneous that it could readily be beaten or rolled out into plate. The preparations of gold that could be readily used in the usual manner were unfit for fillings inserted by the Herbst method. There was something radically different in the method and manner of consolidation.

The morning session of Friday was mainly occupied by the election of officers and the selection of a place for the next meeting. After a number of ballots, Minneapolis was finally chosen.

The election for officers resulted as follows :

*President*—Dr. J. N. Crouse.

*First Vice-President*—Dr. M. W. Foster.

*Second Vice-President*—Dr. C. F. Rich.

*Recording Secretary*—Dr. Geo. H. Cushing.

*Corresponding Secretary*—Dr. A. W. Harlan.

*Treasurer*—Dr. Geo. W. Keely.

*Members of Executive Committee*—Drs. A. O. Hunt, L. D. Shepard, and C. N. Pierce.

ANATOMY, HISTOLOGY, AND MICROSCOPY.

Section V was called. Dr. M. L. Rhein, the Chairman, presented a verbal report, in which he complimented the paper read by Dr. J. L. Williams, on Wednesday evening. Some points in the paper were discussed by Drs. Abbott, Pierce, and Spaulding, after which the Society adjourned to meet in Minneapolis, the first Tuesday in August, 1885.

